# Upadacitinib and its role in the treatment of vitiligo: A new possible therapeutic perspective

**DOI:** 10.1016/j.jdcr.2024.01.033

**Published:** 2024-02-21

**Authors:** Jorge Magdaleno-Tapial, Pablo Hernández-Bel, Altea Esteve-Martínez, Rodrigo Peñuelas-Leal, Carolina Labrandero-Hoyos, José Luis Sánchez-Carazo, Amparo Pérez-Ferriols

**Affiliations:** Department of Dermatology, Hospital General Universitario de Valencia, Valencia, Spain

**Keywords:** case series, JAK, repigmentation, upadacitinib, vitiligo, Vitiligo Extent Score

Vitiligo, a chronic skin disorder characterized by the loss of melanocytes, affects approximately 0.5% to 2% of the global population.^1^ Despite its prevalence, the pathogenesis of vitiligo remains not completely understood. It is believed to be a complex disease involving genetic predisposition, environmental triggers, and autoinflammatory responses leading to melanocyte destruction.^2^ The management of vitiligo has been a challenge because of the unpredictable course of the disease and the variable response to treatment. Traditional therapies have shown some efficacy but are often associated with side effects and inconsistent results.

Recent advances in our understanding of the molecular mechanisms underlying vitiligo have opened new avenues for treatment. Among these, Janus kinase (JAK) inhibitors have emerged as a promising therapeutic approach. JAKs are a family of tyrosine kinases that play a crucial role in the signaling of various cytokines and growth factors implicated in immune responses and inflammation. Dysregulation of JAK signaling pathways has been implicated in various autoinflammatory diseases. In the context of vitiligo, JAK1 has been found to play a significant role in the pathogenesis of the disease.^3^^,^^4^ Upadacitinib, a second-generation JAK inhibitor highly selective for JAK1, is currently approved for the treatment of several immunological diseases, including atopic dermatitis. Until now, 1 case report has been published about the efficacy of upadacitinib in the treatment of vitiligo.^5^

This is a retrospective unicentric case series where patients with nonsegmental vitiligo were treated with upadacitinib. The primary outcome measure was the change in the Vitiligo Extent Score (VES) from baseline to the time of follow-up, 12 or 24 weeks. In all cases, a basic blood test was conducted, and active infections were ruled out as indicated by the drug’s technical data sheet.

Ten patients affected by nonsegmental vitiligo were treated with upadacitinib during a maximum of 24 weeks (Supplementary Table I, available via Mendeley at https://data.mendeley.com/datasets/fjvjfp7k7w/1). Upadacitinib dose was 30 mg daily, with the exception of a 15-year-old patient who was prescribed 15 mg. Mean time of duration of the pathology was 13.7 years. Forty percent of the patients presented with comorbidities, predominantly of an autoinflammatory nature. Only 1 patient was receiving other adjuvant treatment (phototherapy) at the time of starting upadacitinib and none applied topical treatment with corticosteroids or calcineurin inhibitors. No patient had a history of latent tuberculosis infection.

The average baseline VES was 28.6 and mean body surface area was 33% (range 6-70). In general, 90% of the patients showed improvement in the VES after treatment with upadacitinib. All patients with 12-week follow-up showed repigmentation (VES 12-week 24.7). Of the 7 patients with 24-week follow-up (baseline mean VES 36), all but 1 showed repigmentation of the affected area (mean VES 24-week 26). The effectiveness in terms of VES25 or VES50 (improvement of 25% or 50%, respectively, compared with baseline VES) was not statistically significantly associated with the duration of the disease, gender, patient age, or the extent of vitiligo. There was no significant or serious adverse event. One patient developed a herpes gingivostomatitis due to herpes simplex virus-1 after 1 month of treatment that did not require discontinuation of upadacitinib. One patient developed elbow carbuncle after tropical insect bit, which was managed with oral antibiotherapy and suspension of the drug for 1 week. Nine out of 10 patients were satisfied with the treatment and they continue with it (Fig 1).

**Fig 1:**
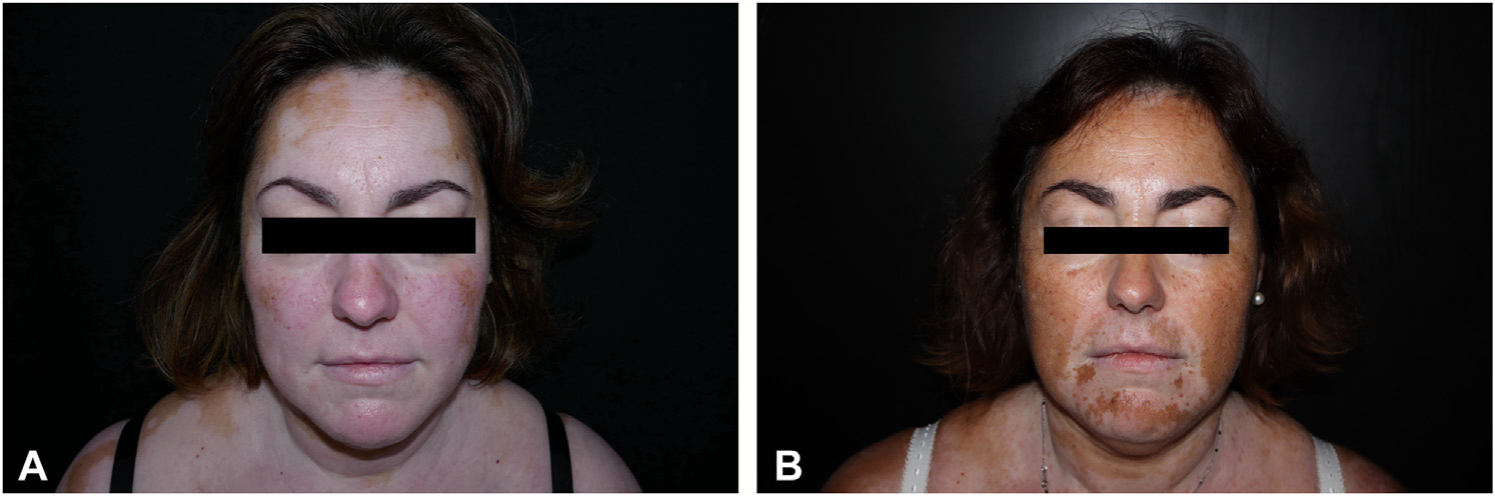
Repigmentation of 80% of the facial region after 36 weeks of treatment with upadacitinib.

Our case series adds to the growing body of evidence supporting the use of upadacitinib, a selective JAK1 inhibitor, in the treatment of vitiligo. In all cases, patients demonstrated significant repigmentation in short-term, suggesting a potential role for upadacitinib in halting disease progression and promoting the restoration of skin color.

The study is limited by its small sample size, the short follow-up of patients and lack of a control group. Although the results are promising, they should be interpreted with caution. Larger, controlled studies are needed to confirm these findings and to further investigate the long-term efficacy and safety of upadacitinib in patients with vitiligo.

In conclusion, to our knowledge, this is the first series of patients with vitiligo treated with upadacitinib. The observed repigmentation in all patients suggests that upadacitinib could be a promising therapeutic option for this challenging condition. However, further research is needed to confirm these findings and to fully elucidate the role of JAK inhibitors in the management of vitiligo.

## Conflicts of interest

None disclosed.
